# Retinopathy of Prematurity (ROP): Are We There Yet?

**DOI:** 10.3390/medicina62050869

**Published:** 2026-05-01

**Authors:** Eva Coughlin, Waylon Alvarado, Veluchamy A. Barathi, Ramani Ramchandran, Deborah M. Costakos, Aparna Ramasubramanian, Shyam S. Chaurasia

**Affiliations:** 1 Ocular Immunology and Angiogenesis Lab, Department of Ophthalmology and Visual Sciences, Medical College of Wisconsin, Milwaukee, WI 53226, USA; ecoughlin@mcw.edu (E.C.); walvarado@mcw.edu (W.A.); dcostakos@mcw.edu (D.M.C.); aramasubramanian@mcw.edu (A.R.); 2 Translational Pre-Clinical Model Platform, Singapore Eye Research Institute, Singapore 169856, Singapore; amutha.b.veluchamy@seri.com.sg; 3 Division of Neonatology, Developmental Vascular Biology Program, Department of Pediatrics, Children’s Research Institute (CRI), Medical College of Wisconsin, Milwaukee, WI 53226, USA; rramchan@mce.edu; 4 Pediatric Ophthalmology, Children’s Wisconsin, Medical College of Wisconsin, Milwaukee, WI 53226, USA; 5 Cell Biology, Neurology and Anatomy Department, Medical College of Wisconsin, Milwaukee, WI 53226, USA

**Keywords:** retinopathy of prematurity, retinal vasculature, anti-Vascular endothelial growth factor, preterm birth, management, vision impairment

## Abstract

Retinopathy of Prematurity (ROP) affects preterm infants worldwide, involving abnormal development of retinal blood vessels associated with supplemental oxygen use in neonatal care. Although there have been strides in identifying at-risk infants, implementing early screening, updating disease criteria through the International Classification of Retinopathy of Prematurity (ICROP), and developing new therapies, ROP remains a leading cause of preventable blindness. As preterm birth survival rates rise, the incidence of ROP continues to increase and is projected to rise even in countries with abundant resources and well-established care programs. Improving ROP care requires global standardization of screening, diagnosis, and management to prevent missed diagnoses and minimize outcome variability. Intravitreal anti-vascular endothelial growth factor (VEGF) injections are changing the landscape of ROP management, but longitudinal research is needed to determine their long-term safety in preterm infants. Effective ROP management relies on teamwork across disciplines and open communication with parents. Given that parents are lifelong caregivers of a child who may be affected by ROP-related vision impairment, including them in the care team and encouraging psychosocial support is vital. Socioeconomic disparities and limited access to ROP-trained ophthalmologists exacerbate disease burden, underscoring the need for innovative solutions to improve access to care. This perspective emphasizes the importance of globally standardizing ROP prevention and care, noting that efforts are still incomplete, equitable access has not been realized, and the long-term role of anti-VEGF agents in ROP treatment remains unclear.

## 1. History of ROP and Epidemiology

**Figure 1:**
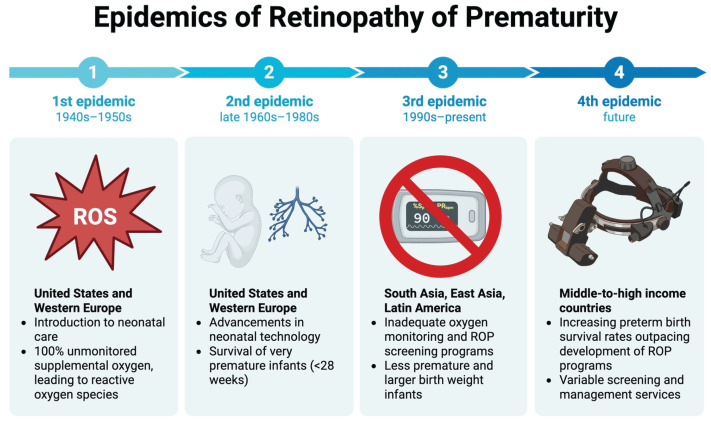
The schematic illustrating the timeline of Retinopathy of Prematurity (ROP) epidemics, affected geographic areas, and key contributing factors worldwide. Created with Biorender.com.

In 1942, an article in the American Journal of Ophthalmology focused on an emerging disease characterized by an abnormal retinal vasculature in prematurely born infants. Dr. Theodore Terry first described this disease, which he termed retrolental fibroplasia (RLF), now known as Retinopathy of Prematurity (ROP). By the 1950s, the prevalence of ROP had increased substantially, becoming the leading cause of childhood blindness in the U.S. [1] and playing a key role in the first epidemic of ROP observed in developed countries [2]. Studies performed in the 1950s by Patz established a direct link between the free use of high oxygen concentrations and the development of ROP. Therefore, restrictions were placed on high, unregulated supplemental oxygen use in preterm infants, which reduced ROP but were accompanied by increased mortality [1]. Determining optimal oxygen conditions to maximize survival while minimizing ROP incidence remains a persistent challenge. The second epidemic of ROP in developed countries occurred from the late 1960s to the 1980s, secondary to advances in neonatal care, permitting the survival of extremely preterm, low birth weight infants [3]. This second wave led to the realization that oxygen was not the sole factor driving ROP development. To define the screening and diagnosis, the International Classification of Retinopathy of Prematurity (ICROP) was established in 1984. The Multicenter Trial of Cryotherapy for Retinopathy of Prematurity (CRYO-ROP) was performed in 1988, establishing cryotherapy as the first treatment to preserve vision in ROP [4]. Currently, low- and middle-income countries (LMICs) are experiencing the third epidemic of ROP due to increased survival rates of preterm infants, inadequate ROP screening and treatment systems, along with a shortage of trained ROP specialists to handle this burden [3]. Addressing this third epidemic poses a unique challenge, requiring innovative solutions to reduce global healthcare disparities. Looking ahead, a fourth epidemic of ROP is predicted to affect middle-to-high income countries, where screening and management services will be overwhelmed by increased survival rates of preterm infants [5]. The timeline of ROP epidemics is illustrated in Figure 1. By critically reflecting on current limitations in ROP screening, diagnosis, and management, there is an opportunity to strengthen care systems and mitigate the impact of this emerging epidemic. Figure 1 outlines the timeline of the four ROP epidemics, the geographic regions affected, and the key contributing factors.

Over 31.9% show no meaningful decrease over time. Approximately 15 million premature infants (<37 weeks’ gestational age) are born each year, and the survival of premature infants with lower gestational age is increasing [6]. Taken together, the persistently high prevalence of ROP and the substantial population of premature infants emphasize that ROP is a significant global issue. Examining the U.S. specifically, the percentage of premature infants diagnosed with ROP increased from 4.4% in 2003 to 8.1% in 2019, which may reflect higher survival rates of extremely low-birth-weight infants due to advances in neonatal care. However, this rise may also be partially influenced by updates to ROP screening guidelines during this period, which increased detection [7]. Regardless of the underlying cause, this surge has increased demand for ROP care, straining healthcare systems and underscoring the need for intervention.

Building on these trends, cohort data from high-income countries reveal differences in both the overall burden of ROP and the proportion of infants who progress to treatment-requiring disease compared with LMICs. The Postnatal Growth and Retinopathy of Prematurity retrospective cohort study, conducted in the U.S. and Canada from January 2006 to December 2011, found that 43.1% of screened infants developed ROP, and 6.9% required treatment. Importantly, treatment-requiring ROP occurred almost exclusively among extremely premature infants [8]. In contrast, LMICs experiencing the third epidemic of ROP, particularly Asian countries, are seeing higher proportions of treatment-requiring disease and have less access to timely treatment [9]. For example, a prospective cohort study conducted in South India in 2018 reported that 32.6% of screened infants developed ROP at any stage, with 13.2% requiring treatment [10]. In essence, income-related disparities in healthcare access amplify the rates of preventable visual impairment due to ROP.

## 2. Pathophysiology

Retinal vascular development begins with vasculogenesis at the optic head around 12 weeks in utero and continues from the center to the periphery until 22 weeks. Post-vasculogenesis, new blood vessels sprout via angiogenesis, primarily driven by vascular endothelial growth factor (VEGF), which is stimulated by the hypoxic environment in the fetus in utero. Complete vascular development of the peripheral retina is not complete until about 40–44 weeks of postmenstrual age [4]. When premature infants are exposed to supplemental oxygen (a hyperoxic environment) in the neonatal intensive care unit (NICU), VEGF levels decrease, prematurely halting normal vascular growth and causing regression of existing vessels [11], often described as the first phase of ROP of vaso-obliteration. The second phase of ROP begins at about 32–34 weeks of postmenstrual age and is characterized by ischemia of the avascular retina, which triggers late VEGF production. If the area of avascular retina is large enough, it can lead to neovascularization [12].

## 3. Risk Factors

The most well-recognized risk factors for ROP include younger gestational age, low birth weight, and supplemental oxygen use [4]. Higher oxygen concentrations, longer durations of use, and fluctuations in oxygen levels have been documented to increase the risk. However, the ideal standards for oxygen use remain under debate. The Surfactant, Positive Pressure, and Pulse Oximetry Trial (SUPPORT) compared lower (85–89%) and higher (91–95%) static oxygen targets, demonstrating the trade-off between increased mortality at lower saturations and increased incidence of ROP at higher saturations [13]. In response, biphasic oxygen strategies have been proposed, targeting oxygen saturation of 85–92% before 34 weeks’ corrected gestational age and >95% thereafter to align with the 2-step hypothesis of neovascularization. Although the results are encouraging, with decreased incidence and severity of ROP without increased mortality, further investigation is needed before this approach can be adopted as a standard NICU practice [14]. Other risk factors discussed include nutrition, medications, genetics, demographics, and maternal, prenatal, and perinatal factors. For example, hypertensive disorders of pregnancy, such as preeclampsia and eclampsia, are associated with higher levels of antiangiogenic factors, and studies have found an increased risk of ROP with these conditions [15].

## 4. Screening and Diagnosis

Screening is essential in identifying premature infants at risk for severe ROP and managing them promptly to prevent subsequent vision loss. Screening guidelines for ROP are continually evolving and vary by country. In the U.S., ROP screening is recommended for infants with a gestational age (GA) ≤ 30 weeks, a birth weight (BW) ≤ 1500 g, or infants with a BW between 1500 and 2000 g or a GA > 30 weeks who have an unstable clinical course and the clinical judgment of a neonatologist for risk of ROP [16]. If an infant meets any one of these three criteria, it is recommended that they be screened. Several more restrictive criteria have been considered to reduce the screening burden, but ultimately, broader criteria, including both GA and BW, have been retained to ensure no treatable ROP cases are missed. For example, a GA-only criterion of <32 weeks was discussed following an international study of 136,546 infants conducted in the UK and other countries with comparable neonatal mortality rates, which found that no infant with a GA > 30 weeks required ROP treatment. However, this approach was rejected because there were infants with GA > 32 weeks but very low BW who still developed ROP and required treatment. Therefore, relying on GA alone would risk missing small-for-gestational-age infants. Additionally, more restrictive BW criteria (<1251 g) have been considered, given that most severe cases occur below this threshold, yet a few infants in the 1251–1500 g range still develop treatable disease [17].

The UK ROP screening guidelines are similar to those in the U.S., recommending screening at <31 weeks GA or <1501 g BW, which is fairly consistent across high-income countries. In contrast, screening criteria vary substantially worldwide, depending on socioeconomic context, with lower-income countries often using broader thresholds. For example, the screening guidelines in India are GA ≤ 34 weeks or BW ≤ 2000 g [18]. These higher GA and BW cutoffs are due to variability in neonatal care and to higher rates of severe, earlier-onset ROP in more mature infants. While necessary to avoid missing at-risk infants, broader screening criteria significantly increase the number of infants requiring screening, thereby straining already limited workforce capacity and ROP systems of care. This global variability highlights that ROP screening criteria are shaped by resource availability. Despite advances in ROP services, disparities in resources across countries prevent the adoption of universally standardized screening criteria, suggesting we have not yet reached an optimal approach to ROP prevention and care.

The initial screening is scheduled at 4 weeks postnatal age, or 31 weeks postmenstrual age, whichever is later [19]. The examination involves dilating the pupil and performing binocular indirect ophthalmoscopy, with a lid speculum and scleral depression as needed, to visualize the retina. The screening examination is to be performed by an ophthalmologist trained in ROP, who can utilize ICROP to classify, diagram, and record retinal findings [12].

Telemedicine is also being integrated to expand access to screening via TeleROP. TeleROP uses wide-angle retinal imaging in the NICU, enabling images to be sent to remote ROP experts for analysis and expanding access to care in rural and underserved areas. A notable example of TeleROP is the Stanford University Network for Diagnosis of Retinopathy of Prematurity (SUNDROP) telemedicine initiative, which involved ROP specialists remotely evaluating retinal images from infants across six neonatal intensive care units between 2005 and 2010. The program demonstrated 100% sensitivity and 100% negative predictive value for detecting treatment-warranted ROP, providing evidence that TeleROP is accurate and clinically reliable [20]. A TeleROP study in Southern India revealed how telemedicine can improve access to ROP screening in underserved areas while reducing socioeconomic barriers to care. The Retinopathy of Prematurity—Save Our Sight (ROPE-SOS) program sent trained technicians in mobile units equipped with retinal cameras to rural areas, where images were captured and transmitted to ROP experts at the Aravind Eye Institute [21].

**Figure 2:**
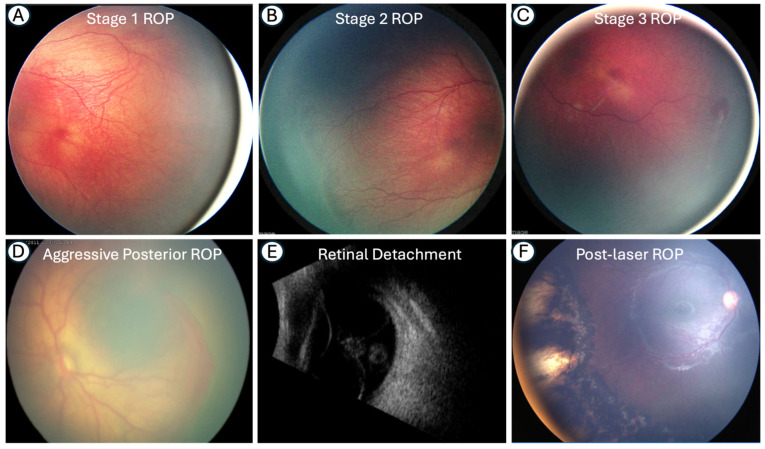
Clinical images demonstrating the progression of ROP through stages 1–3 (**A**–**C**), as well as A-ROP (**D**) and retinal detachment on ultrasound (**E**), along with a post-laser treatment image (**F**).

According to the third edition of ICROP, published in 2021, ROP should be classified by zone, plus disease, stage, and extent. In cases with aggressive ROP (A-ROP), this should also be documented [22]. Figure 2 describes the clinical diagnosis for different stages of ROP.

## 5. Management

It is important to note that the management of infants with ROP differs based on disease severity. Routine examinations with an ophthalmologist and diligent use of supplemental oxygen are common for all infants, but interventional treatment options are recommended once the disease has met the criteria for ‘Type 1 ROP’. These include disease presence in: Zone I with Stage 3 severity, Zone I with Stage 1 or 2 severity with plus disease, or Zone II with Stage 2 or 3 with plus disease [4].

Cryotherapy, which uses externally applied freezing temperatures to ablate retinal tissue, was the primary treatment for ROP and works by inducing scarring in the avascular retina, thereby preventing further vascular growth [16]. In the early 1990s, laser photocoagulation replaced cryotherapy and remains a mainstay for ROP treatment, achieving regression rates of approximately 90% [4]. Compared with cryotherapy, laser therapy is associated with less post-treatment pain, reduced disruption of the blood–retinal barrier, fewer systemic complications, and improved visual outcomes. Laser photocoagulation ablates the avascular retina using a diode laser to prevent pathologic neovascularization [23]. However, the pitfall of this approach is that it can cause permanent retinal damage and preclude subsequent vascularization of the peripheral retina.

To address these limitations and promote retinal preservation and physiological vascular development, anti-VEGF therapies were introduced in 2007. Administered via intravitreal injection, anti-VEGF agents target the second phase of ROP by inhibiting angiogenesis. Monoclonal antibodies such as bevacizumab and ranibizumab are among the first-line anti-VEGF therapies. However, their use is considered “off-label” for both. Aflibercept is the only anti-VEGF agent recently approved by the U.S. Food and Drug Administration (FDA) for the management of ROP. These medications can be used in conjunction with laser photocoagulation or as monotherapy. Additionally, certain studies have reported lower rates of myopia with anti-VEGF therapy compared to laser treatment, suggesting improved visual outcomes with newer treatment strategies [24].

The BEAT-ROP trial (Bevacizumab Eliminates the Angiogenic Threat of ROP), conducted from 2008 to 2010, was the first randomized controlled trial comparing intravitreal bevacizumab monotherapy with conventional laser photocoagulation for ROP treatment. This study represented a shift in the ROP treatment paradigm, demonstrating that bevacizumab monotherapy was more effective than laser for stage 3+ zone I disease, but not for zone II disease [25]. Safety concerns about systemic VEGF suppression prompted an investigation into dose-reduction strategies. In 2017, a study found that 5% of the dose used in BEAT-ROP achieved treatment success in 9 of 9 eyes, suggesting that lower doses may retain therapeutic efficacy while potentially mitigating systemic risk [26]. From 2015 to 2017, the RAINBOW (Ranibizumab Compared with Laser Therapy for the Treatment of Infants Born Prematurely with ROP) study compared the efficacy and safety of two ranibizumab doses with conventional laser therapy, and, like BEAT-ROP, demonstrated improved ocular outcomes with anti-VEGF. Additionally, RAINBOW reported a short-term safety profile for ranibizumab with no changes in systemic VEGF levels [27]. Lastly, the FIREFLEYE Randomized Clinical Trial (Effect of Intravitreal Aflibercept vs. Laser Photocoagulation on Treatment Success of Retinopathy of Prematurity) evaluated the utility of aflibercept vs. laser therapy and found that aflibercept achieved the treatment success in 85.5% of cases compared to 82.1% for laser treatment and did not meet criteria for noninferiority [28]. Collectively, these studies have established intravitreal anti-VEGF therapy as a new mainstay for ROP treatment, while also emphasizing the continued need for research to elucidate the most effective use of these therapies.

Furthermore, additional research is required to elucidate the long-term systemic safety of anti-VEGF therapy in premature infants, particularly considering concerns that systemic VEGF suppression might contribute to neurodevelopmental delays [4]. Results from the RAINBOW extension study are encouraging, demonstrating that neurodevelopmental outcomes at 2 years were similar between infants treated with ranibizumab and those treated with laser photocoagulation [29]. Despite these reassuring findings, there are other clinical considerations with anti-VEGF therapies. A meta-analysis comparing anti-VEGF therapy with laser photocoagulation found that anti-VEGF agents were associated with a higher risk of recurrence, necessitating prolonged follow-up, and that bevacizumab was associated with a longer retreatment interval [30]. Thus, ongoing research focuses on optimizing dosing strategies for anti-VEGF agents to reduce recurrence and maximize injection intervals.

There is no global consensus on which anti-VEGF agent to use as first-line therapy, and agents are typically selected based on the ophthalmologist’s experience and preferences. The American Academy of Pediatric Ophthalmology and Strabismus (AAPOS) does not provide official recommendations on which anti-VEGF agent to use, but rather indicates that the three aforementioned anti-VEGF agents can be used in conjunction with laser therapy or as monotherapy [31]. Bevacizumab remains the most commonly used agent in the U.S., despite aflibercept’s FDA approval in 2023, because of its history, physician familiarity, and overall effectiveness. Ranibizumab has been approved for use in ROP treatment in Europe [32]. Laser photocoagulation (with or without anti-VEGF therapy) remains the recommended first-line treatment modality as more research is gathered on the definitive role of anti-VEGF agents [33].

Management of infants with ROP requires intense follow-up and re-evaluation for treatment needs. Follow-up intervals vary among infants, depending on disease severity and progression. The ICROP provides specific guidance on how often examinations should be performed during both the acute management stages and when infants transition to less frequent check-in visits [22].

## 6. Parental Perspective

ROP management requires multidisciplinary collaboration between ophthalmologists, neonatologists, neonatal nurses, and other healthcare professionals. Parents should also be regarded as essential members of this care team, as they are responsible for making time-sensitive decisions on behalf of their baby and for serving as lifelong caregivers for a child who may experience vision loss related to ROP. Clear and empathetic communication between the healthcare team and parents is essential for parents to feel equipped with the knowledge to care for their child and supported. Ophthalmologists must communicate with parents with sensitivity, acknowledging that ROP may develop during an infant’s recovery from other health issues [34]. The screening process itself is anxiety-inducing for parents, to such an extent that there has been controversy concerning whether parents should accompany their infant during screening [35]. However, the presence of parents during screening strengthens involvement in the care process and creates an emotionally stable environment that can comfort the baby. This may also increase trust and confidence in the treating doctor, improving adherence to follow-up visits [36]. Ultimately, transparent communication with parents about what to expect during the screening process will facilitate informed decision-making. A balance between promoting meaningful integration of parents into the care team, protecting parental emotional well-being, and honoring individual coping styles is perhaps ideal.

Given the limited data on caregivers’ experiences during ROP screening and follow-up, a study at the University of California, Los Angeles (UCLA) Medical Centers surveyed caregivers to assess the impact on families. The study found that caregivers of children with ROP experienced long-term family impact compared to those of children who were screened but did not have the disease [37]. Many of the children with ROP in this study were under the care of more than one type of eye subspecialist. This indicates that caregiver support extends beyond ROP alone and includes management of other ophthalmic complications. A similar study in India found that ROP adversely affected the vision-related quality of life of children and their parents, particularly in terms of family impact and concerns about their child’s future. Quality of life declined with increasing disease severity and the occurrence of ocular sequelae of ROP [38]. Together, these studies highlight the significant burden of ROP on families in both developed and developing countries, underscoring the need for improved support resources to help caregivers navigate long-term ophthalmic care and associated psychosocial stressors. Parents often feel isolated. Therefore, connecting with peer support groups to openly share their fears and concerns can build a sense of community and provide opportunities to learn from others with similar experiences.

## 7. Ongoing Challenges and Future Directions

In 2021, ROP-related blindness was most prevalent in low and low-middle-SDI (Socio-Demographic Index) countries, where access to neonatal and ophthalmic care is limited. Broader socioeconomic factors, including lower education levels, inadequate insurance coverage, higher out-of-pocket healthcare costs, and nursing staff shortages, were associated with higher rates of visual impairment [5]. A shortage of healthcare providers, particularly ROP-trained ophthalmologists who are disproportionately concentrated in urban areas, further exacerbates disparities in rural populations. ROP is not confined to resource-limited areas and remains a significant concern in high-income countries as well. In 2018, ROP was identified as the most common treatable cause of childhood blindness in the U.S [39]. Increasing survival rates of preterm infants, a reflection of advances in neonatal care, are outpacing screening and management capacity. This has contributed to rising trends in ROP-related vision loss across high-middle and middle-SDI regions since 2005 and supports the projection of a fourth emerging ROP epidemic [5]. Anticipating this ROP surge, improving global consensus on ROP screening and diagnostic guidelines, and addressing socioeconomic and geographic disparities should be of the utmost priority.

ROP is a global disease warranting sustained attention and continued advancement in detection and management strategies. Future directions in ROP care must adapt to meet the demands of a growing population of premature infants who are born at the limits of viability and with increased disease burden. Nanopremature and micropremature infants are significantly more likely to develop treatment-requiring ROP [40]. Imaging innovations enable earlier detection of pathologic changes, helping ensure timely treatment and prevent progression to severe disease. For example, peripheral optical coherence tomography (OCT), assisted by scleral depression, has emerged as a valuable tool for enhancing visualization of the peripheral retina [41]. Additionally, artificial intelligence platforms such as ROPtool can be utilized to address variability in disease classification. ROPtool measures retinal vascular tortuosity and width to quantify a plus disease score and determine whether plus disease is present, a key determinant of ROP treatment [42]. This approach of promoting diagnostic consistency also supports telemedicine-based screening models. Global implementation of TeleROP has the potential to improve equitable access to ROP care, facilitate earlier detection, and reduce the number of cases that progress to diseases requiring treatment.

Finally, anti-VEGF therapies are highly effective for initial disease regression but are complicated by disease reactivation when retinal vascularization is incomplete. Reactivation is currently managed with repeat intravitreal anti-VEGF injections and prophylactic laser treatment of the avascular anterior retina. This requires long-term follow-up until vascularization is complete. Further, the long-term consequences of anti-VEGF therapy during infancy are not known. Our ongoing research focuses on identifying new targets, conducting future studies to refine dosing to reduce reactivation, improving retreatment strategies, and developing models for prolonged surveillance [43]. Ultimately, the future of ROP will depend on technological integration, diagnostic standardization, and optimized treatment strategies to meet the needs of an increasingly vulnerable global infant population.

## Data Availability

The data supporting this article will be made available by the authors on request.

## Abbreviations

The following abbreviations are used in this manuscript:

ROP	Retinopathy of Prematurity
ICROP	International Classification of Retinopathy of Prematurity
VEGF	Vascular Endothelial Growth Factor
RLF	Retrolental Fibroplasia
U.S.	United States
CRYO-ROP	Cryotherapy for ROP
LMICs	Low- and Middle-Income Countries
NICU	Neonatal Intensive Care Unit
SUPPORT	Surfactant, Positive Pressure, and Pulse Oximetry Trial
IGF-1	Insulin-like Growth Factor
WINROP	Weight, IGF-1, Neonatal ROP
GA	Gestational Age
BA	Birth Weight
UK	United Kingdom
TeleROP	Telemedicine in ROP
SUNDROP	Stanford University Network for Diagnosis of ROP
ROPE-SOS	ROP-Save Our Sight
FDA	Food and Drug Administration
BEAT-ROP	Bevacizumab Eliminates the Angiogenic Threat of ROP
RAINBOW	Ranibizumab Compared with Laser Therapy for the Treatment of Infants Born Prematurely with ROP
AAPOS	American Association for Pediatric Ophthalmology and Strabismus
UCLA	University of California Los Angeles
SDI	Socio-Demographic Index
OCT	Optical Coherence Tomography
